# Novel Technique for Management of Axillary Hidradenitis Suppurativa Using Setons

**DOI:** 10.1155/2015/369657

**Published:** 2015-08-02

**Authors:** Sepehr Seyed Lajevardi, Jayantha Abeysinghe

**Affiliations:** Department of Surgery, Canterbury Hospital, 575 Canterbury Road, Campsie, NSW 2194, Australia

## Abstract

Management of hidradenitis suppurativa (HS) of the axilla which is nonresponding to conservative management presents a significant therapeutic challenge. Most surgical treatment options are associated with significant morbidities and prolonged hospital stay. We present a technique of management of HS using setons which is simple and allows the ongoing treatment to be done on an outpatient basis. Given the fact that HS is a chronic relapsing condition each recurrence may again be managed using this technique. This will allow the patients to manage their recurrences with minimal impact on their activities of daily living.

## 1. Introduction

Hidradenitis suppurativa (HS) is a suppurative relapsing inflammatory disease that involves the apocrine glands of the skin [1]. It commonly occurs in the axilla with formation of multiple abscesses. Conservative treatment options include local hygiene, weight loss, oral and topical antibiotics and steroids, and immune suppressive therapy [2]. Various surgical techniques have been proposed for the management of HS that is nonresponding to conservative measures. Incision and drainage of individual abscesses are often ineffective in gaining control of the disease given the persistence of underlying sinus tracts. Wide local excision and healing with secondary intention with or without the use of negative pressure therapy, or with closure using local cutaneous flaps or skin grafts, are the most effective method of treatment [1–3]; however, this is associated with significant morbidity and is often associated with prolonged in-patient hospital stay. These techniques are often unsuccessful to control the disease and some are associated with significant morbidity and prolonged hospitalization. Placement of setons has been used widely for management of perianal fistula tracts with great success [4]. In this report we present a case of chronic HS which was nonresponding to conservative management that was successfully treated with placement of multiple setons.

## 2. Case Presentation

**Figure 1:**
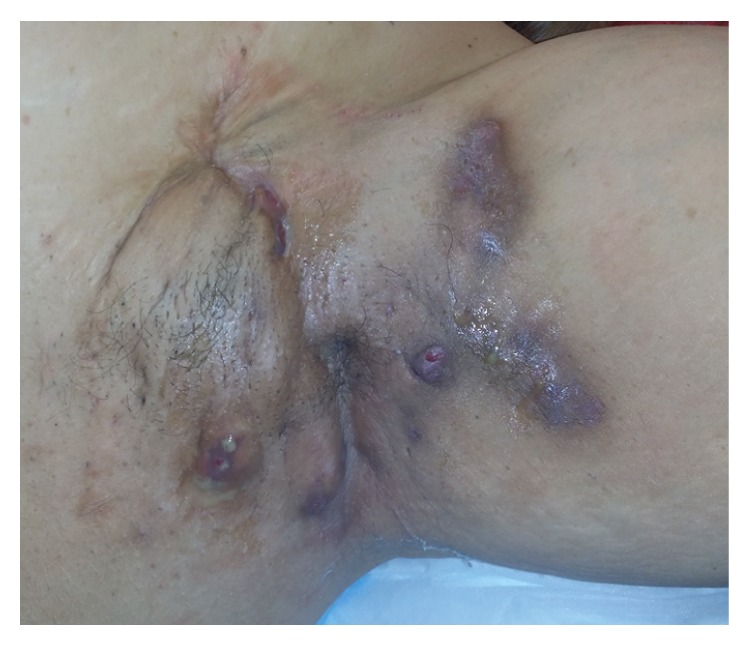
Image of the left axilla on presentation showing multiple abscess formation with pus discharge and surrounding induration, inflammation, and scarring.

A 48-year-old female was referred to our institution with more than 20-year history of recurrent HS affecting both axillae and groins. Her background medical history was only significant for hypertension stabilized with ace-inhibitor therapy. She maintained meticulous hygiene of the affected regions and had been previously treated with oral and topical antibiotics and steroids. Despite such treatment her disease has been progressively worsening involving a wider area and more frequent episodes of abscess formation and cellulitis. She has had frequent presentations to the hospital for treatment with intravenous antibiotics and incision and drainage of abscesses. On examination she was found to have a BMI of 30. The left axilla showed severe HS with multiple abscesses discharging pus with surrounding induration and scarring (Figure 1). The other involved areas were not acutely infected.

She was taken to the operating theatre and each abscess was drained and the cavity was explored using a probe. This revealed multiple subcutaneous fistula tracts connecting the abscess cavities. After irrigation with hydrogen peroxide and 0.9% normal saline, multiple setons were placed in each of the fistula tracts. The wounds were covered with absorptive dressing. She was discharged home the following day with continuing oral antibiotics treatment with twice-daily Amoxicillin 875 mg/Clavulanic Acid 125 mg.

**Figure 2:**
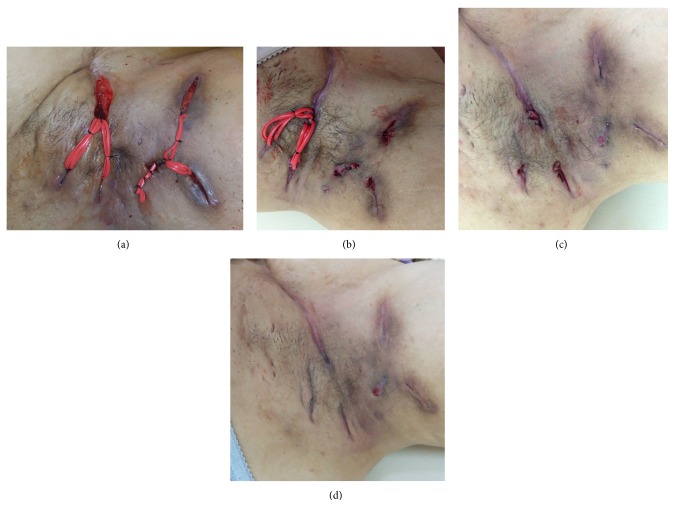
(a) Day 0 left axilla postoperatively showing five fistula tracts with setons insertion. ((b), (c), and (d)) Showing the progress of the left axilla at 4, 6, and 8 weeks postoperatively, respectively, and showing healing of all wounds and complete resolution of the inflammation.

Swabs taken during the operation revealed growth of* Streptococcus milleri*. Over the following 2 weeks the wounds showed significant reduction in inflammation and antibiotics were ceased. By 4 weeks a number of the wounds had improved significantly and the setons were removed from these. By 6 weeks all wound had improved and all remaining setons were removed. By 8 weeks there was complete resolution of inflammation and abscess formation in the left axilla (Figure 2). Subsequent follow-up at 6 months revealed the lack of recurrence in the treated area.

## 3. Discussion

Surgical treatment of HS with wide local excision and secondary reconstruction is challenging and is often associated with significant morbidity and prolonged hospital stay. Given the fact that HS is often a chronic disease, we believe the treatment plan is based on controlling the episodes of recurrence of infection with minimum intervention. In this way the disease may be managed with minimal impact on the patient's activities of daily living through short hospital stay and outpatient review of the wounds. The treatment with setons allowed our patient to be discharged from the hospital the day after the operation and the outpatient follow-up only involved second weekly review by the treating specialist. The setons used are the same as those used for management of perianal fistulae.

Golcman et al. [1] point out the etiology of chronic HS to be due to the presence of distant cutaneous orifices interconnected through a subcutaneous fistula and call them “bridging lesions.” Therefore, the authors propose a surgical technique of subcutaneous fistulectomy to remove these tracts. Our technique of identifying these “bridging lesions” using a probe and inserting a seton to keep them open is more simple surgically and more time efficient and comes with less pain and surgical morbidity to the patient given the fact that no tissue has been excised. The seton will keep the tract open much similar to their application in the perianal fistula [4]. Allowing drainage will lead to subsidement of the active infection and chronic inflammation. Subsequently the removal of the setons once inflammation has reduced allows these tracts to heal and our experience shows that there was no need for fistulotomy. Given the simplicity of this technique, we believe it could be repeated in the cases of disease recurrence and that may be preferable to many patients compared to the morbidity associated with wide local excision and reconstruction of the entire diseased area. Based on our literature review this is the first report of the use of setons for management of HS in the axilla.

Surgical management of axillary HS using setons is safe and simple and may be beneficial compared to other proposed surgical techniques for disease which is nonresponding to conservative measures.
